# Occipital anaplastic oligodendroglioma with multiple organ metastases after a short clinical course: a case report and literature review

**DOI:** 10.1186/1746-1596-9-17

**Published:** 2014-01-21

**Authors:** Gang Li, Zhiguo Zhang, Jianghong Zhang, Tianbo Jin, Hongjuan Liang, Li Gong, Guangbin Cui, Haixia Yang, Shiming He, Yongsheng Zhang, Guodong Gao

**Affiliations:** 1 Department of Neurosurgery, Tangdu Hospital, Fourth Military Medical University, Xi’an 710038, P.R China; 2 National Engineering Research Center for Miniaturized Detection Systems, School of Life Sciences, Northwest University, Xi’an 710069, P.R China; 3 Department of Pathology, Tangdu Hospital, Fourth Military Medical University, Xi’an 710038, P.R China; 4 Department of Radiology, Tangdu Hospital, Fourth Military Medical University, Xi’an 710038, P.R China; 5 Department of Administrative, Tangdu Hospital, Fourth Military Medical University, Xi'an 710038, P.R. China

**Keywords:** Oligodendroglioma, Extracranial metastasis, Autopsy, 1p/19q, Chromosome deletions, Genetic analysis

## Abstract

**Background:**

It is generally believed that malignant gliomas never metastasize outside the central nervous system (CNS). However, the notion that oligodendrogliomas (OGDs) cells cannot spread outside CNS is being challenged.

**Methods:**

We described in detail the clinical story of one patient with anaplastic OGD, which metastasized to lymph nodes, bone marrowand bones Genetic analyses included detection of 1p and 19q chromosomal arms, methylation status of *MGMT* promoter, and *PTEN* exon mutations. A search of worldwide literature was conducted for reports of metastatic OGDs using NCBI-PubMed, with the keywords “extracranial”, “extraneural”, “oligodendroglioma”, “oligodendrogliomas”, “metastatic”, “metastasis”, and “metastases”, in different combinations.

**Results:**

An open biopsy of the infiltrated bones in our patient revealed that malignant cells had replaced the patient’s marrow. Moreover, the diagnosis of multiple-organ metastases of anaplastic OGD was confirmed based on immunohistochemical staining. Genetic analyses showed that the tumors originated from previously resected brain lesions. None of the lesions had 1p and 19q deletions, but hypermethylation of *MGMT* promoter, and the G → A transversion at codon 234 of *PTEN* exon 2 were detected. Literatures review yielded 60 reports of metastatic OGDs from 1951 to the present, which with our patient makes 61 cases. Concerning these 61 patients, there were 110 infiltrated sites correlated closely with primary OGDs. The most frequent metastatic sites were bone and bone marrow (*n* = 47; 42.7%), lymph nodes (*n* = 22; 20.0%), liver (*n* = 7; 6.4%), scalp (*n* = 6; 5.5%), lung (*n* = 6; 5.5%), pleura (*n* = 4; 3.6%), chest wall (*n* = 3; 2.7%), iliopsoas muscle (*n* = 2; 1.8%), soft tissue (*n* = 2; 1.8%), and parotid gland (*n* = 2; 1.8%).

**Conclusions:**

Extracranial metastases in anaplastic OGD are very rare but they do occur; bone and bone marrow may be the most common sites. Detection of certain molecular markers such as deletion of 1p and 19q chromosomal arms, hypermethylation of *MGMT* promoter, and characteristic *PTEN* exon mutations may help differentiate subtypes which are more prone to extracranial metastases.

**Virtual slides:**

The virtual slide(s) for this article can be found here: http://www.diagnosticpathology.diagnomx.eu/vs/8749838611478560.

## Background

It has been widely believed by neurosurgeons and neurooncologists that malignant gliomas never metastasize outside the central nervous system (CNS) [1]. However, this notion has gradually proved incorrect. A review of 8000 tumors involving the CNS found only 35 cases of extracranial metastasis, including one oligodendroglioma (OGD) [2]. Liwnicz and Rubinstein [3] analyzed 116 cases in the literature and found that the most common metastasizing tumor type was glioblastoma multiforme (41.4%) followed by medulloblastoma (26.7%), ependymoma (16.4%), and astrocytoma (10.3%). and OGD (5.25%) was the least common tumor type to metastasize [3,4].

OGD is an uncommon diffuse glial tumor of central neuroepithelial origin, accounting for ~4.2% of all primary brain tumors. It has been mostly identified in adults, with the highest incidence occurring in the fifth and sixth decades of life, although it has also been reported in children and adolescents [5]. Various forms of combination therapy administered as comprehensive treatment have improved the survival of patients with OGD or mixed oligoastrocytoma [6].

Herein, we report a case of anaplastic oligodendroglioma (AO) that metastasized to multiple lymph nodes, bone marrow, and bones, including the bilateral iliac bones, the right acetabulum, and multi-vertebral bodies.

## Case presentation

**Figure 1:**
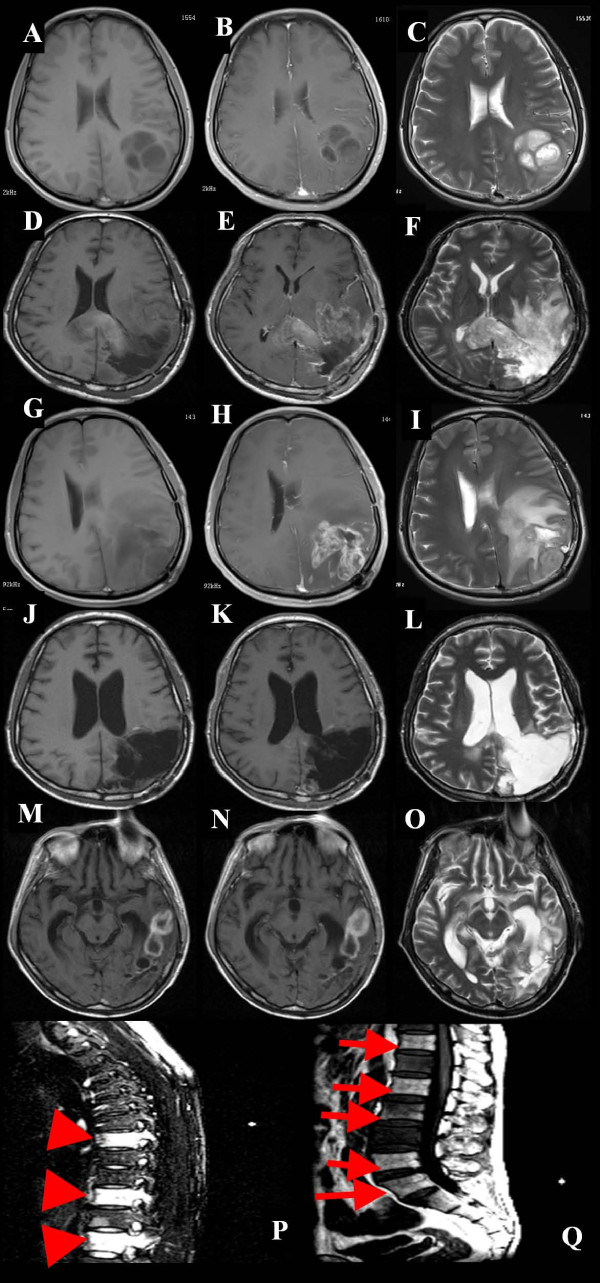
**Representative axial MR images with gadolinium, taken on initial admission. (A)** T_1_-weighted MR image (T_1_WI); **(B)** Contrast-enhanced T_1_WI and **(C)** T_2_-weighted MR image (T_2_WI) showing a left occipital tumor with mild contrast enhancing; **(D)** at one month follow up, T_1_WI; **(E)** contrast-enhanced T_1_WI and **(F)** T_2_WI showing no apparent enhanced lesion; **(G)** 8 months later after the first surgery, T_1_WI; **(H)** contrast-enhanced T_1_WI and **(I)** T_2_WI showing marked enhanced mass on the cavity and recurrence; **(J)** 48 h after the second surgery, T_1_WI; **(K)** contrast-enhanced T_1_WI, and **(L)** T_2_WI showing no apparent enhanced lesion; **(M)** 8 months later after combination radiotherapy and chemotherapy, T_1_WI; **(N)** contrast-enhanced T_1_WI and **(O)** T_2_WI showing marked enhanced mass of the wall in the posterior portion of the removal cavity and left temporal areas, and recurrence of the enhanced tumor. **(P, Q)** 8 months later after combination radiotherapy and chemotherapy, sagittal spinal contrast-enhanced T_1_WI after gadolinium infusion showing high-intensity mass lesion in **(P)** T7, T10, and T12 vertebral bodies (arrowheads) and **(Q)** T12, L2, L3, L5, and S1 vertebral bodies (arrows).

**Figure 2:**
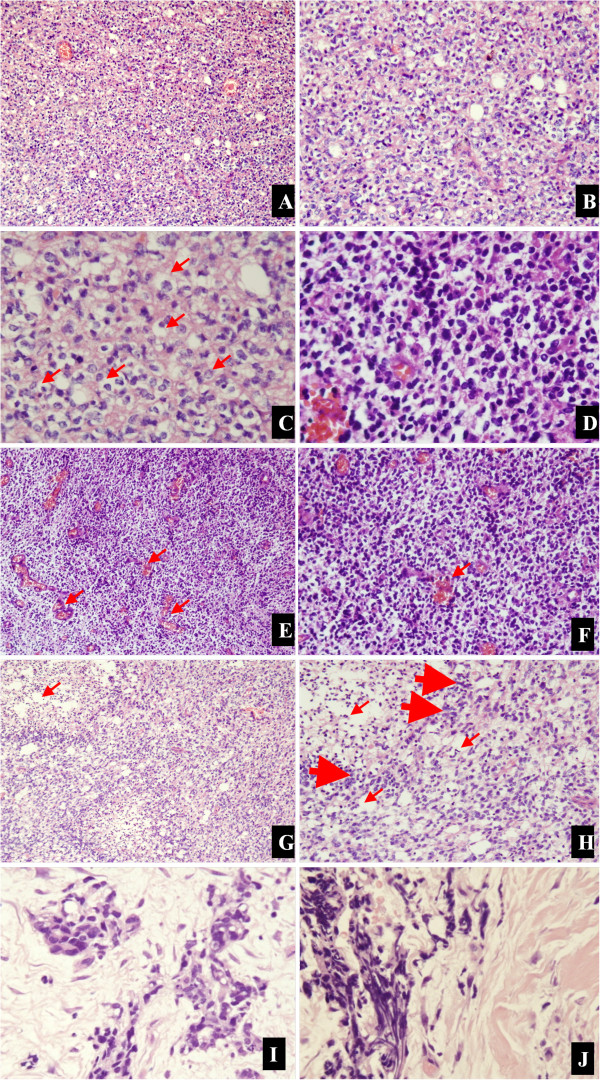
**Representative photomicrographs of the tumor specimens.** Higher cell densities (**A**, **B**; 100×, 200×, respectively, H & E) with perinuclear haloes (**C**; 200×, H & E; arrows), and microscopically round-to-oblong cells with hyperchromatism and pleomorphism (**D**; 400×, **H & E**), are compatible with AO. **(E, F)** Clusters of capillary or plexiform capillaries (arrows), and the irregular mitosis densities were higher (100×, 200×, respectively, **H & E**). **(G, H)** Obvious false fence structure (thick arrows)-shaped necrosis (slim arrows; 100×, 200×, respectively, **H & E)**. **(I, J)** After the right iliac bone marrow needle biopsy, cells in the bone marrow specimen from the patient were small and round with a thin rim of eosinophilic cytoplasm (400×, H & E).

A 45-year-old male who initially presented with a short history of headache and vomiting was admitted to our hospital in September 2011. No focal neurological deficit was found on admission. Magnetic resonance imaging (MRI) showed a left solid occipital tumor with mild contrast enhancing (Figure 1A,B,C). On 15 September 2011 he underwent a left solid occipital craniotomy with gross total resection confirmed by subsequent MRI scans (Figure 1D,E,F). The mass was yellow and friable. It was neither hemorrhagic nor necrotic. The tumor margin was ill defined. Photomicrographs of the resected tumor showed that there were higher cell densities, densely packed round cells with perinuclear haloes, microscopically round-to-oblong cells with hyperchromatism and pleomorphism (Figure 2A-D), clusters of capillary or plexiform capillaries (Figure 2E,F), and obvious false fence structure-shaped necrosis (Figure 2G,H). In addition, the irregular mitosis densities were higher. A diagnosis was made of AO, WHO (World Health Organization) grade III [7,8]. During the subsequent 6 months, he was given 4 cycles of adjuvant chemotherapy with temozolomide (TMZ; Schering, NJ), a standard regimen [9] of 150–200 mg · m^-2^ · d^-1^ for 5 days, repeated every 28 days.

A repeated left occipital tumor resection was performed 8 months later (Figure 1G-L). Pathology also showed AO WHO grade III, with similar histology. Subsequently, irradiation therapy concomitant with TMZ, 75 mg · m^-2^ · d^-1^ for 42 days, was given and then 3 cycles of adjuvant chemotherapy with a dose-intensive regimen of TMZ [10] of 75 mg · m^-2^ · d^-1^ for 21 days, repeated every 28 days. The patient experienced no significant hematological toxicity, but he came to have difficulties in understanding and remembering.

In October 2012, 5 months after the final occipital resection, he presented with a 3-week history of lumbar and right hipbone pain, and was hospitalized again in November 2012. Regretfully, brain MRI showed evident progression of the intracranial lesion (Figure 1M-O). There was a solid enhancing lesion of high signal intensity on T_2_-weighted MR images, and low-intensity signals in the temporal area. The new lesion was thought to be a recurrent tumor with malignant transformation (Figure 1M-O). MR images of the spine showed diffuse patchy areas of increased signal intensity and abnormal enhancement of the T7, T10, T12, L2, L3, L5, and S1 vertebral bodies (Figure 1P,Q).

**Figure 3:**
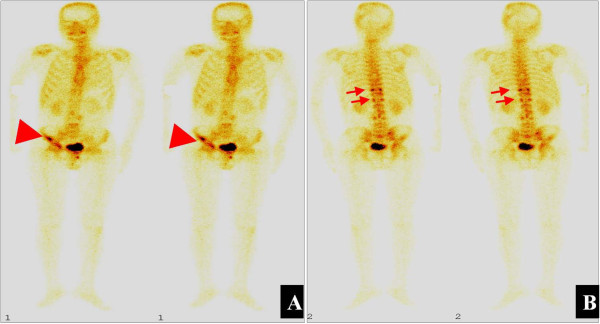
**Representative emission computed tomography scans. (A)** anterior scans, and **(B)** posterior scans, showed a hypermetabolically abnormal uptake at the right iliac bone (arrowheads) and T10, and T12 vertebral bodies (arrows).

**Figure 4:**
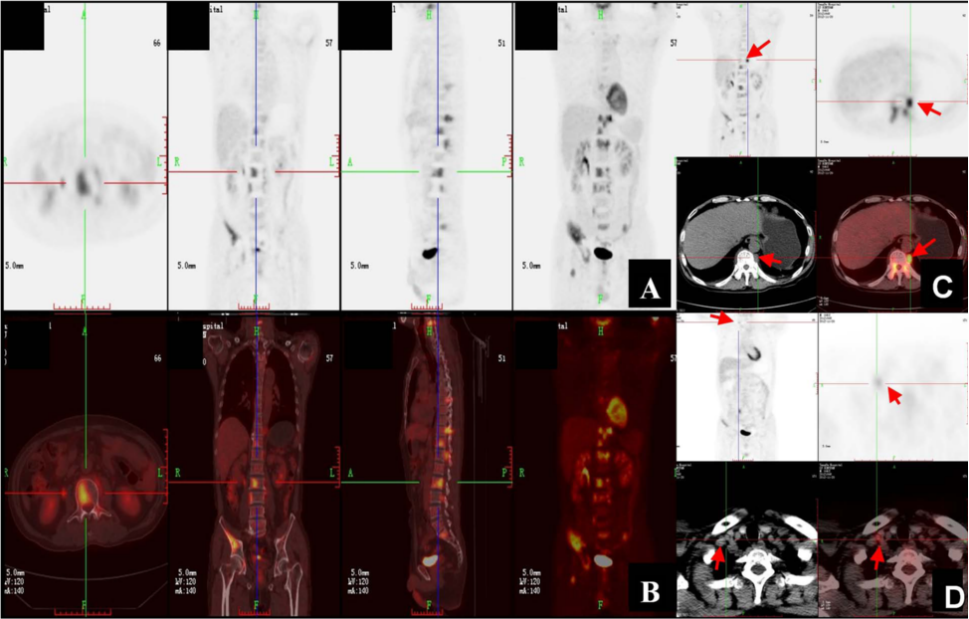
**Representative whole body PET**-**CT scans. (A)** and **(B)** Multiple foci of increased 18 F-fluoro-2-deoxyglucose (FDG) uptake at the bilateral iliac bones, C4, T7, T11, T10, T12, L2, L3, S1 vertebral bodies and the right acetabulum; **(C)** Focus of increased ^18^ F-FDG uptake at the lymph node near the left side of the T11 vertebral body (arrows); **(D)** Focus of increased ^18^ F-FDG uptake at the lymph node of the right supraclavicular region (arrows).

Subsequent bone scintigraphy and positron emission tomography (PET)-computed tomography (CT) scans revealed more multifocal invasion. A whole body ^99m^Tc-methylene diphosphonate bone scan showed hyper-activity in the right iliac bone and the tenth and twelfth thoracic vertebral bodies (Figure 3). PET-CT scans also showed multifocal invasion of the bilateral iliac bones, the right acetabulum, the right femoral neck and the C4, T7, T10, T11, T12, L2, L3, and S1 vertebral bodies (Figure 4A,B), the lymph nodes at the left side of the eleventh thoracic vertebral body (Figure 4C) and the right supraclavicular region (Figure 4D).

**Figure 5:**
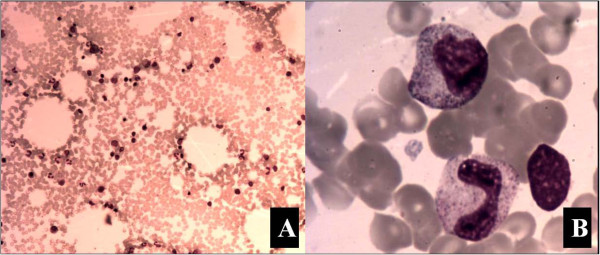
**Representative scans of bone marrow smear. (A, B)** No plasmocytoma cells were found (100× and 1000×, respectively).

**Figure 6:**
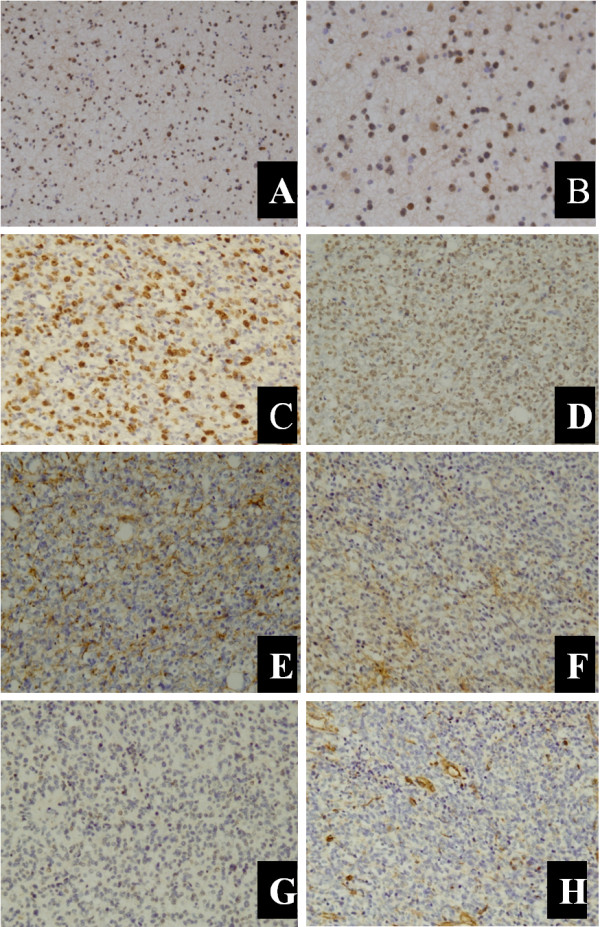
**Representative immunochemical markers in tumor specimens. (A, B)** Positive reaction for IDH1 (100×, 200×, respectively). **(C)** Positive reaction for Ki-67, with proliferating index >80% (200×). **(D, E)** Tumor cells were positive for GFAP and Oligo-2, respectively (200×). **(F, G, H)** showed the tumor cells were negative for EMA, MGMT, and vimentin, respectively (200×).

A bone marrow smear of the right iliac bone showed no plasmacytoma cells (Figure 5). An open biopsy of the infiltrated right iliac bone revealed the replacement of the patient’s marrow by malignant cells, which exhibited nuclear pleomorphism (Figure 2I,J). The confirmed diagnosis of AO metastasis to bone marrow was based on immunohistochemical staining. We simultaneously reviewed and performed the tumor cell identification in the bone marrow spaces as well as in the previously primary AO in the brain. They were all strongly positive for isocitrate dehydrogenase-1 (IDH1; Figure 6A,B) and Ki-67, with proliferation index >80% (Figure 6C). They were also all positive for the glial fibrillary acidic protein (GFAP) marker (Figure 6D), which is positive in glial, Schwannian, and ependymal tumors (all neural tumors), and for the marker oligodendrocyte transcription factor (Oligo-2; Figure 6E). These findings supported the CNS origin of the metastatic cells. Further findings were all negative for other pertinent immunohistochemical stains: epithelial membrane antigen (EMA), O6– methylguanine-DNA methyltransferase (MGMT) and vimentin (Figure 6F,G,H, respectively). Multiple outside pathologists confirmed this diagnosis. After discussion with the patient and his family, he was admitted to the family ward and supportive care was administered until his death on 19 January 2013.

## Materials and methods

### Tissue samples

We collected and analyzed both the bone marrow from the above mentioned open biopsy and the brain tumor tissues resected in the first and second craniotomy. The Human Research Committee of the Fourth Military Medical University approved the use of human tissues, in accordance with the Approval of Research Involving Human Subjects. The patient’s custodians provided informed written consent. All specimens were handled and made anonymous in accordance with ethical and legal standards.

### DNA extraction

The bone marrow from the biopsy was decalcified using ultrasonic decalcification with ethylenediaminetetraacetic acid (EDTA) as described previously [11] and paraffin embedded. DNA was extracted from the tumor tissues as previously described [12,13]. In brief, DNA was isolated by sodium dodecyl sulfate/proteinase K treatment, phenol–chloroform extraction, and ethanol precipitation and then dissolved and stored in 1× Tris-EDTA (TE) buffer. DNA concentration was measured at optical density 260 (OD_260_), and purity was verified by OD_260_/OD_280_ ≈ 1.8.

### Fluorescent in situ hybridization (FISH) assay

Sections from the brain tumors and metastatic lesions were evaluated with routine hematoxylin-eosin (H & E) staining. FISH assays were also performed for molecular characterization. Both the brain tumors and the metastatic lesions were analyzed to perform dual-color FISH assay as previously published [14]. Briefly, paired probes for 1p36/1q25-q31 and 19q13/19p12 (Abbott Molecular) were prepared [14]. Green and red fluorescent signals were enumerated under a Leica microscope with appropriate filters. For each hybridization, a minimum of 40 non-overlapping nuclei were assessed for numbers of green and red signals, counted separately by two individuals.

### MGMT promoter methylation status

DNA from the tumor tissues was analyzed by performing a methylation-specific polymerase chain reaction (MS-PCR) assay, as previously published [15], to detect the status of the *MGMT* promoter.

### PCR single-strand conformation polymorphism (PCR-SSCP) and DNA sequencing for phosphatase and tensin homologue (PTEN) deleted on chromosome ten exons

PCR–SSCP assay was applied to elucidate the mutations in exons 1–9 of the *PTEN* gene (Table 1) [16-18]. PCR amplification was carried out in a final volume of 25 μL containing 50 ng DNA, 2.5 μL of 10× PCR buffer, 1.5 mmol/L MgCl_2_, 10 pmol/L of each primer, 2.5 mmol/L of each dNTP, and 1 U Taq DNA polymerase. The amplification conditions were: an initial incubation at 95°C for 8 min; 35 cycles at 95°C for 30 s, 51–57°C for 45 s for each primer specific to the *PTEN* exons (Table 1), and 72°C for 30 s; with a final extension at 72°C for 7 min. PCR products were resolved in 2% agarose gels stained with ethidium bromide with a 100-bp DNA ladder as a standard reference, and electrophoresed for 30 min at 100 V.

**Table 1:** ***PTEN *** **primers used for single**-**strand conformation polymorphism** (**SSCP**) **analysis**

**Exon**	**Primer sequences**	**Annealing temp. (°C)**	**Amplicon (bp)**
1	F1. 5’-TCCTCCTTTTTCTTCAGCCAC-3’	56	147
	R1. 5’-GAAAGGTAAAGAGGAGCAGCC-3’		
2	F2. 5’-TGCATATTTCAGATATTTCTTTCCTT-3’	57	155
	R2. 5’-TTTGAAATAGAAAATCAAAGCATTC-3’		
3	F3. 5’-TGTTAATGGTGGCTTTTTG-3’	56	114
	R3. 5’-GCAAGCATACAAATAAGAAAAC-3’		
4	F4. 5’-TTCCTAAGTGCAAAAGATAAC-3’	56	147
	R4. 5’-TACAGTCTATCGGGTTTAAGT-3’		
5	F5. 5’-TTTTTTTTTCTTATTCTGAGGTTATC-3’	51	312
	R5. 5’-GAAGAGGAAAGGAAAAACATC-3’		
6	F6. 5’-AGTGAAATAACTATAATGGAACA-3’	54	231
	R6. 5’-GAAGGATGAGAATTTCAAGC-3’		
7	F7. 5’-ATCGTTTTTGACAGTTTG-3’	55	262
	R7. 5’-TCCCAATGAAAGTAAAGTAGA-3’		
8	F8. 5’-AGGTGACAGATTTTCTTTTTTA-3’	52	394
	R8. 5’-TAGCTGTACTCCTAGAATTA-3’		
9	F9. 5’-CTTTCTCTAGGTFAAGCTGTACTT-3’	55	231
	R9. 5’-TTCATGGTGTTTTATCCCTCTTGA-3’		

SSCP analyses of all *PTEN* exons were conducted systematically on the PCR products. Equal volumes (7 μL) of the PCR products and loading buffer (95% formamide, 20 mM EDTA, 0.05% bromphenol blue, and 0.05% xylene cyanol) were mixed and centrifuged for 15 s, heat-denatured at 95°C for 7 min, snap-chilled on ice for 10 min, and resolved through an 8% non-denaturing polyacrylamide gel (acrylamide to bisacrylamide, 29:1) containing 50 mM Tris-borate (pH 7.5) and 2.5 mM EDTA, and electrophoresed with 1× tris-borate-EDTA buffer for 16 h at 14°C at a voltage of 100 V. Silver staining was performed as previously described [19].

According to the PCR-SSCP results of genomic DNA, the present sample was considered PCR-SSCP positive based on the evident difference in the single strand strip number and electrophoresis transference location [20]. Genomic DNA from the positive PCR-SSCP sample was amplified again in a 40-μL reaction system for bidirectional DNA sequencing. The amplified PCR products were sequenced with an ABI PRISM 310 dye terminator cycle sequencing ready reaction kit. The results were compared using the GenBank database.

## Results

### FISH analysis

**Figure 7:**
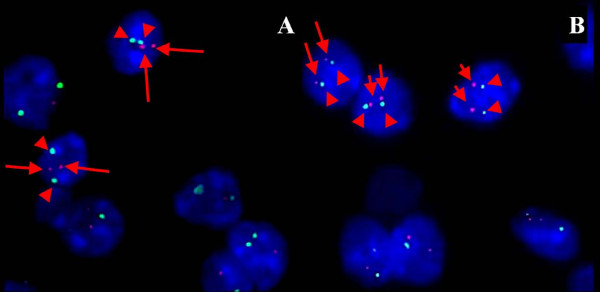
**Representative FISH images from the right iliac bone.** Some signals are missing due to nuclear truncation. **(A)** No 1p deletion, with 2 red (1p36, arrows) and 2 green (1q25-q31, arrowheads) signals in scattered nuclei. **(B)** No 19q deletion with 2 red (19q13, arrows) and 2 green (19p12, arrowheads) signals in scattered nuclei.

None of the tumors from the resected brain lesions or the metastatic lesions had the 1p (Figure 7A) or 19q deletions (Figure 7B). We were satisfied that, although the bone marrow from the biopsy was decalcified via ultrasonic decalcification and EDTA, the metastatic lesions remained informative.

### Methylation status of the MGMT promoter

**Figure 8:**
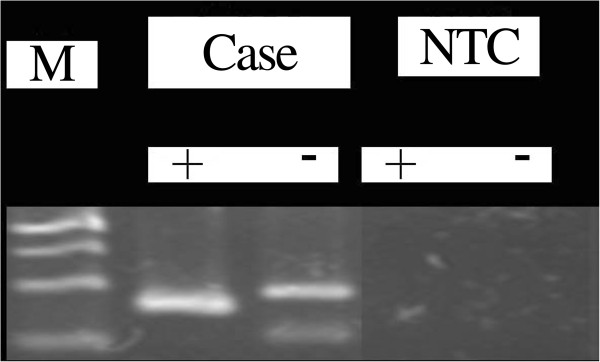
**Representative MSP**-**PCR for methylated *MGMT *promoter.** NTC, non DNA template control; +, methylated; –, unmethylated; M, DNA marker; the upper, middle, and lower bands are 140, 120, 100, and 80 bp, respectively.

The MSP-PCR assays of the tumors from the primary brain lesions and autopsied metastasized tissues all showed the methylated *MGMT* promoter (Figure 8), and the result from the corresponding samples was nearly identical.

### PTEN mutation

**Figure 9:**
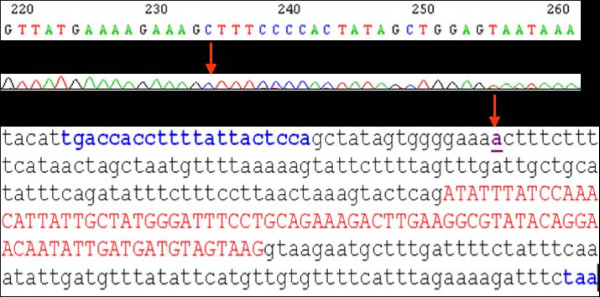
**Representative sequencing data showing the G** → **A transversion at codon 234 (arrows) of exon 2 in *PTEN *of the patient.** Blue, primer; red, exon 2; purple, the transversion of A, showing the polymorphism.

The case was considered PCR-SSCP-positive, and genomic DNA from the samples was amplified for bidirectional DNA sequencing. Compared with the sequences in the GenBank database, the sequencing data of the patient showed a transversion, “A” in place of “G” at codon 234 of exon 2. This was thought to be a likely single nucleotide polymorphism (Figure 9). No mutation was detected in the other *PTEN* exons.

## Discussion

Extraneural metastases from primary brain tumors are rare [21] for reasons that remain obscure. Proposed initial theories generally lack credibility among neurosurgeons and neurooncologists; these include collapse of thin-walled cerebral veins, the inability of neural tissue to grow outside the CNS, and the lack of lymphatics in the brain [22]. More accepted is the theory that because brain tumors present earlier, there is less time for metastases to develop [21]. Another theory suggests that the intracerebral environment is not sufficiently hostile to select out metastatic clones. There is relatively little connective tissue stroma in the brain compared with the rest of the body. It has also been proposed that clones are not selected for the ability to invade fibrous connective tissue and are thus not suited to invade extracranial tissues [23]. The role of the blood–brain barrier is also uncertain, albeit it does seem to have a limited role in metastasis.

Despite the above theories, glioma does metastasize outside the CNS, and most cases of extraneural metastasis (nearly 96%) have occurred after surgical excision of the primary tumor [24]. The most common glioma to metastasize is glioblastoma multiforme, followed by medulloblastoma and ependymoma [4], while OGD metastasizes very rarely.

OGD is such a diffuse glial tumor. Extracranial metastasis of the primary intracranial neoplasm is infrequent generally, and it seemed to usually happen only in the setting of prior neurosurgical resection [25]. OGDs are characterized by multiple recurrences [25], extraneural spread is unusual, and distant skeletal metastases are particularly infrequent. We therefore undertook a worldwide literature review to investigate further the incidence of extraneural metastases. This yielded 60 previously reported metastatic OGDs from 1951 to the present, and our patient makes 61 (Table 2). The review was performed using NCBI-PubMed with the keywords “extracranial”, “extraneural”, “oligodendroglioma”, “oligodendrogliomas”, “metastatic”, “metastasis”, and “metastases” in different combinations. We also reviewed the bibliography of the subsequently selected articles.

**Table 2:** Review from the literature of 61 reported patients with oligodendroglioma with metastases outside the CNS

**Patient no.**	**Gender**	**Subjects**	**WHO grade**	**Age at diagnosis (y)**	**Location of metastasis**	**OS (months)**	**Time between presentation of primary tumor and metastasis (months)**	**Molecular biomarkers tested**	**Reference**
1	F	British	ODG, low grade early, later *AO	25	Cervical lymph nodes, scalp, lung, bone	84	*	*	James et al. 1951 [26]
2	F	American	ODG, *grade	7	Scalp, bone, soft tissue, liver	36	*	*	Spataro et al. 1968 [27]
3	F	Austrian	ODG, *grade	58	Bone	30	*	*	Jellinger et al. 1969 [28], (also reported as Schuster et al. 1976 [29])
4	M	American	*ODG, *grade	45	Bone marrow	17	*	*	Smith et al. 1969 [2]
5	*	*	ODG, initially low grade	3.5	Lymph nodes, lungs, adrenal	21	*	*	Kernohan 1971 [30]
6	F	Scottish	Malignant glioma, some ODG features	21	Bone	9	*	*	Eade et al. 1971 [31]
7	M	Scottish	Small cell GBM with regions of ODG	23	Bone	12	*	*	
8	M	French	*ODG, *grade	57	Lymph nodes, bone	20	*	*	Cappellaere et al. 1972 [32]
9	M	French	*ODG, *grade	22	Lymph nodes, bone, parotid gland	25	*	*	
10	F	British	Malignant glioma, some ODG features	30	Pleura	156	*	*	Brander et al. 1975 [33]
11	M	German	*ODG, *grade	40	Bone, lymph nodes, lungs	86	*	*	Kummer et al. 1977 [34]
12	*	Canadian	*ODG, *grade	*	Scalp	*	*	*	Chin et al. 1980 [35]
13	F	American	ODG, grade II	33	Bone, lymph nodes, scalp, soft tissue	50	*	*	Ordonez et al. 1981 [36]
14	M	Japanese	Grade III	32	Bone	76	14	*	Nakamura et al. 1985 [37]
15	M	*	ODG, malignant	41	Bone marrow	48	*	*	Newman et al. 1985 [38]
16	*	Canadian	ODG, *grade, some astro	12	Bone, lymph nodes, scalp	104	*	*	Macdonald et al. 1989 [39]
17	*	Canadian	ODG, *grade	44	Cervical lymph nodes, bone,	48	*	*	
18	F	Canadian	ODG, grade III	36	Bone, lymph nodes	60	*	*	
19	*	Canadian	ODG, *grade, some astro	32	Bone, lymph nodes, scalp	38	*	*	
20	*	Canadian	ODG, *grade	34	Bone	76+	*	*	
21	M	Canadian	ODG, grade III	27	Bone	37	*	*	
22	*	Canadian	ODG, *grade, some astro	47	Bone	26+	*	*	
23	*	Polish	Grade III	*	*	*	*	*	Rolski et al. 1993 [40]
24	*	German	*	*	Cervical lymph nodes	*	*	*	Steininger et al. 1993 [41]
25	M	British	ODG, grade III	54	Bone marrow	12	*	*	Gerrard et al. 1995 [42]
26	*	German	Grade III	*	Cervical lymph nodes	*	48	*	Schroder et al. 1995 [43]
27	M	Italian	ODG, grade II	58	Chest wall, bone marrow, possibly liver	48	*	*	Monzani et al. 1996 [44]
28	M	British	ODG, grade III	43	Bone marrow, liver	3	*	*	Dawson 1997 [45]
29	M	Austrian	ODG-astrocytoma grade III	62	Thoracic wall, pleura, bone marrow	288	6	*	Finsterer et al. 1998 [46]
30	M	Indian	Grade III	50	Bone and bone marrow	7+	*	*	Anand et al. 2001 [47]
31	*	Italian	ODG, grade II	25	Bone	84+	*	*	Giordana et al. 2002 [48]
32	M	Indian	Grade III	50	Bone marrow	17	*	*	Sharma et al. 2003 [49]
33	M	American	Grade III	33	Bone marrow	38+	*	*	Choon et al. 2004 [50]
34	M	Canadian	Grade III	35	Bone	84	23	Allelic LOH of chromosome 1p (1p-), a rise in serum alkaline phosphatase	Morrison et al. 2004 [51]
35	F	American	ODG, grade II early, later AO, grade III	41	Parotid gland	79+	68	Codeletion of 1p/19q	Wang et al. 2004 [52]
36	M	American	ODG, low grade early, later AO, grade III	28	Bone marrow	120	5	Positive for S-100 marker with immunohistochemical stain	Al-Ali et al. 2005 [23]
37	M	American	Atypical meningioma, early, later AO, grade III	32	Bone, bone marrow, cervical and thoracic lesions	168	27	Elevated serum lactate dehydrogenase and alkaline phosphatase, positive for S-100, and GFAP with immunohistochemical stain	Merrell et al. 2006 [53]
38	F	American	AO, grade III	71	Bone, bone marrow,	17	13	Codeletion of 1p/19q, positive for S-100, and GFAP with immunohistochemical stain, elevated serum hemoglobin level	
39	F	Chinese	ODG, *grade	64	Pleura, bone	*	84	Positive for S-100, Olig2 and GFAP with immunohistochemical stain	Lee et al. 2006 [54]
40	M	American	ODG, grade II early, later AO, grade III	15	Pleura, bone, lungs, adrenal gland, chest, liver, abdomen	62	31	Codeletion of 1p/19q	Bruggers et al. 2007 [55]
41	F	Japanese	AO, grade III	17	Spleen, liver, pancreas, bone, spinal dura mater, dorsal root ganglia, lungs, lymph nodes, iliopsoas muscle	144	3	Markedly positive for Ki-67, and positive for Olig2 with immunohistochemical stain,	Uzuka et al. 2007 [56]
42	F	Korean	AO, grade III	48	Liver, lungs	28	27	Not presented	Han et al. 2008 [4]
43	M	Italian	AO, grade III	40	Bone, bone marrow, liver	10	5	Not presented	Zustovich et al. 2008 [57]
44	F	Slovenian	AO, grade III	54	Cervical and neck lymph nodes	48	12	Positive for S-100, and GFAP with immunohistochemical stain	Volavsek et al. 2009 [58]
45	F	Slovenian	AOA, grade III	30	Bone	58+	17	Positive for GFAP with immunohistochemical stain	
46	M	Japanese	AO, grade III	53	Lymph nodes, bone, spinal dura mater, thymus gland, chest wall	30	24	Codeletion of 1p/19q	Noshita N, et al. 2010 [59]
47	F	Japanese	AO, grade III	73	Cervical lesion	18+	18	*	Oshiro S, et al. 2010 [60]
48	M	Dutch	AO, grade III	59	Bone marrow	33	31	Codeletion of 1p/19q, positive for S-100, and p53 with immunohistochemical stain	Krijnen JL, et al. 2010 [61]
49	M	Dutch	*ODG, *grade	24	Bone marrow	*	84	Not presented	
50	M	Dutch	AO, grade III	34	Cervical lymph nodes, iliopsoas muscle	*	not presented	Not presented	
51	M	Dutch	AO, grade III	64	Axillary lymph nodes	*	34	Not presented	
52	M	Dutch	AO, grade III	72	Bone marrow	*	6	Not presented	
53	F	Dutch	*ODG, *grade	67	Bone marrow	*	29	Not presented	
54	M	Dutch	AO, grade III	62	Retroperitoneal lymph nodes	*	21	Not presented	
55	M	Turkish	AO, grade III	55	Bone marrow	23+	11	Positive for GFAP with immunohistochemical stain	Kural C, et al. 2011 [62]
56	M	Chinese	AO, grade III	37	Bone	58+	37	Positive for S-100, and GFAP with immunohistochemical stain	Wu Y, et al. 2011 [63]
57	M	Italian	AO, grade III	40	Bone marrow	61	57	Positive for enolase, and GFAP with immunohistochemical stain	Cordiano V, et al. 2012 [64]
58	F	Turkish	ODG, *grade	*	Cervical lymphatic chain	*	60	Codeletion of 1p/19q	Can B, et al. 2012 [65]
59	M	Turkish	AO, grade III	58	Breast	*	*	*	Alacacioglu A, et al. 2012 [66]
60	M	Irish	AO, grade III	58	Bone	120+	108	Codeletion of 1p/19q, positive for GFAP with immunohistochemical stain	Greene J, et al. 2013 [67]
61	M	Chinese	AO, grade III	45	Bone, bone marrow, lymph nodes	16	13	No deletion of 1p/ 19q, *PTEN* mutation in exon 2, *MGMT* promoter methylated, positive for IDH1, Ki-67, Olig2, and GFAP, negative for MGMT, EMA and Vim with immunohistochemical stain	Present case

F: *f*emale, *M*: male, *OS*: overall survival, *WHO*: World Health Organization, *LOH*: loss of heterozygosity, *ODG*: oligodendroglioma, *AO*: anaplastic oligodendroglioma, *AOA*: anaplastic oligoastrocytoma, *GBM*: glioblastoma multiforme.

*Not provided in the corresponding literature.

+ alive at publication.

Of the 61 reported metastatic OGDs, 33 (54.1%) were male, 17 (27.9%) were female, and in the remaining cases 18.0% gender was not reported (Table 3). Ten (16.4%) patients were Asian, 30 (49.2%) were European, 18 (29.5%) were American or Canadian, and for the remaining 3 (4.9%) the ethnicities were unreported. There were 110 infiltrated sites correlated closely with primary OGDs. The most frequent metastatic site was bone and bone marrow (*n* = 47; 42.7%) followed by lymph nodes (*n* = 22; 20.0%), liver (*n* = 7; 6.4%), scalp (*n* = 6; 5.5%), lung (*n* = 6; 5.5%), pleura (*n* = 4; 3.6%), chest wall (*n* = 3; 2.7%), iliopsoas muscle (*n* = 2; 1.8%), soft tissue (*n* = 2; 1.8%), parotid gland (*n* = 2; 1.8%), and adrenal gland, spleen, thoracic wall, pancreas, dorsal root ganglia, abdomen, spinal dura mater, breast, and thymus gland with one lesion each (*n* = 1; 0.9%).

**Table 3:** Clinical features of 61 patients with extracranial metastatic oligodendrogliomas

	**Cases**	**Rate**
Gender		
Total	61	
Male	33	54.1% (33/61)
Female	17	27.9% (16/61)
Not given	11	18.0% (11/61)
Subjects		
Total	61	
Asian	10	16.4% (10/61)
European	30	49.2% (30/61)
American & Canadian	18	29.5% (18/61)
Not given	3	4.9% (3/61)
Location of metastasis		
Total Sites	110	
Bone/bone marrow	47	42.7% (47/110)
Lymph node	22	20.0% (22/110)
Liver	7	6.4% (7/110)
Scalp	6	5.5% (6/ 110)
Lung	6	5.5% (6/ 110)
Pleura	4	3.6% (4/110)
Chest wall	3	2.7% (3/110)
Iliopsoas muscle	2	1.8% (2/110)
Soft tissue	2	1.8% (2/110)
Parotid gland	2	1.8% (2/110)
Other	9	
Adrenal	1	0.9% (1/110)
Spleen	1	0.9% (1/109)
Thoracic wall	1	0.9% (1/110)
Pancreas	1	0.9% (1/110)
Dorsal root ganglia	1	0.9% (1/110)
Abdomen	1	0.9% (1/110)
Spinal dura mater	1	0.9% (1/110)
Breast	1	0.9% (1/110)
Thymus gland	1	0.9% (1/109)

The review indicated that bone and bone marrow are the most common sites metastasizing from OGDs. In our present case, systematic examination found multiple extracranial metastases, including the vertebrae, lymph nodes, bilateral iliac bones, and right acetabulum. Metastases in these sites suggest that tumor cells were delivered via the blood vessels and lymphatic system.

Primary neoplasm in the brain is generally considered to spread in any of three ways: seeding through the cerebral fluid pathway, local invasion, or spreading remotely through lymphatic and blood vessels [68]. It is widely accepted that the brain and spinal cord contain no lymphatic pathway. However, as the tumor cells infiltrate the dura mater, extracranial metastasis by way of the lymphatic system becomes possible, and could especially happen after craniotomy. Surgical procedures can elevate the risk of metastasis outside the nervous system by way of the lymphatic system as well as the blood vessel.

Extraneural metastasis is considered correlated with multiple craniotomies, shunt surgery, and long-term survival [39,69,70]. Extracranial metastasis without previous surgical intervention is infrequent; among 282 reported cases of glioma with metastases outside the CNS only 24 (8.5%) were spontaneous [71]. Most cases of extracranial metastasis occur after craniotomy. Shunt surgery is responsible for seeding tumor cells by way of cerebrospinal fluid to outside spaces [72]. Prolonged survival might also raise the risk of extracranial metastases. Thus, in our present case, craniotomy could be considered an influencing factor in extracranial metastasis.

The median age of the 60 patients found through the literature review was 40.0 years (range 3.5-73.0 y; Table 2). The overall survival ranged from 3–288 months, with a median of 38 months. These data are consistent with the recent reports of AOs [57,63]. The survival time of our patient was relatively shorter than the median, although he was given the standard regimen recommended by the National Comprehensive Cancer Net (NCCN) guidelines [73]. His shorter survival and poor prognosis may be due, firstly, to the presence of 1p/19q, which may have adversely influenced the success of the recommended comprehensive therapy, as combined deletion of the 1p and 19q chromosomal arms is expected in OGD [74]. Molecular studies have revealed that deletions of chromosome 1p and 19q are usually associated with longer survival in OGD, as well as a better response to irradiation and chemotherapy. Tumors with such a co-deletion are sensitive to comprehensive therapy, with 90-100% of patients responding [74,75]. The overall survival for AOs is about 2–3 years for those without the 1p/19q codeletion, compared with 6–7 years in those with 1p/19q loss [76,77]. For our patient, the previously resected tumors from the brain lesions, as well as the metastatic lesions, all had complete 1p and 19q chromosomes. Thus the lack of deletion of 1p/19q may have led to a shorter survival time under comprehensive therapy.

A second contributing factor toward the poor prognosis of the present case is the presence of the *PTEN* mutation, which was shown in both brain lesions and extracranial metastases. This may have made the patient more prone to extracranial metastases. *PTEN* tumor suppressors are located on human chromosome 10q23.3, which contains nine exons and encodes a 47-kD dual-specific protein phospholipid phosphatase with 403 amino acids [17]. *PTEN* mutations accompany nearly 50% of the cases with a 10q deletion, suggesting that there might be another progression-related target gene in this region [78]. *PTEN* mutations and 10q deletions are more common in AOs without 1p and 19q losses [79]. Infrequently AOs carry activating mutations in the PIK3CA gene [80]. *PTEN* mutations have also been studied for their involvement in the pathogenesis of a number of human malignancies, including glioma [80].

Different mutations in the *PTEN* locus, including frameshifts and missense mutations, have proved to be correlated with human cancers [81,82]. These could result in early termination of translation and immature gene products, and subsequently lead to complete loss of vigor. In most cases, mutations in *PTEN* were found to decrease phosphatase activity [19,83]. In the present study, our patient definitely had a substitution mutation in *PTEN* at exon 2, which may have made him more prone to extracranial metastases.

It must also be noted that, although our patient underwent a chemotherapy regimen with TMZ, his condition nevertheless deteriorated more rapidly afterward. As known, AOs are chemosensitive neoplasms that respond to combined treatment with lomustine, vincristine and procarbazine (i.e., PCV therapy), with 60-70% of patients responding [84]. Studies have also shown that TMZ, the oral alkylating agent that inhibits DNA replication by methylating nucleotide bases, is active and particularly well tolerated in AO patients [85,86]. TMZ methylates guanines in DNA at the O6 position, leading to base-pair mismatch. The known O6– methylguanine (O6–MeG) lesion causes DNA double-strand breaks and subsequent cell death through autophagy, apoptosis, or both [87]. MGMT is the DNA repair enzyme that repairs the O6-MeG lesion and is induced either by chemotherapeutic agents or environmental carcinogens. Methylation of the MGMT promoter or high levels of MGMT are thought to be associated with resistance to TMZ [88]. Levin et al. [86] reported that TMZ was active in patients with progressive OGDs, and that a 1p deletion and low MGMT protein expression could contribute to a better response to TMZ treatment. For our patient, results of the MSP-PCR assays of the primary brain lesions and autopsied metastatic tissues all showed the methylated MGMT promoter. However, we regret that although several cycles of chemotherapy of TMZ were given, the patient still deteriorated rapidly and eventually succumbed. The reasons for his failure to respond favorably to TMZ chemotherapy remain to be explored.

## Conclusions

In summary, extracranial metastases in AO do occur, although they are very rare. Detection of molecular markers such as combined deletion of the 1p and 19q chromosomal arms, hypermethylation of the *MGMT* promoter, and *PTEN* exon mutations may help elucidate which subtypes of AO are more prone to extracranial metastases, which would benefit these patients.

## Consent

Written informed consent was obtained from the patient's family for publication of this Case Report and any accompanying images. A copy of the written consent is available for review by the Editor-in-Chief of this journal.

## Abbreviations

OGD: Oligodendrogliomas; MRI: Magnetic resonance imaging; MGMT: O-6-methylguanine-DNA methyltransferase; PTEN: Phosphatase and tensin homolog; CNS: Central nervous system; AO: Anaplastic oligodendroglioma; WHO: World Health Organization; PET-CT: Positron emission tomography-computed tomography; EDTA: Ethylenediaminetetraacetic acid; IDH1: Isocitrate dehydrogenase-1; GFAP: Glial fibrillary acidic protein; EMA: Epithelial membrane antigen; MS-PCR: Methylation-specific polymerase chain reaction; PCR-SSCP: PCR single-strand conformation polymorphism; FISH: Fluorescent in situ hybridization; NCCN: National comprehensive cancer net.

## Competing interests

The authors declare that they have no competing interest.

## Authors’ contributions

YZ and GG designed the study. GL, ZZ, SH and GC participated in designing and coordinating the study, and GL drafted the manuscript. ZZ and TJ worked together to draft the manuscript. JZ, HL, LG and HY participated in following up the patient, reviewing the relevant articles, and helped to improve the manuscript. All authors have read and approved the final manuscript.
